# Classification of Patients With Sepsis According to Immune Cell Characteristics: A Bioinformatic Analysis of Two Cohort Studies

**DOI:** 10.3389/fmed.2020.598652

**Published:** 2020-12-03

**Authors:** Shi Zhang, Zongsheng Wu, Wei Chang, Feng Liu, Jianfeng Xie, Yi Yang, Haibo Qiu

**Affiliations:** Jiangsu Provincial Key Laboratory of Critical Care Medicine, Department of Critical Care Medicine, Zhongda Hospital, School of Medicine, Southeast University, Nanjing, China

**Keywords:** sepsis, immune status, heterogeneity, endotype, immunoparalysis

## Abstract

**Background:** Sepsis is well-known to alter innate and adaptive immune responses for sustained periods after initiation by an invading pathogen. Identification of immune cell characteristics may shed light on the immune signature of patients with sepsis and further indicate the appropriate immune-modulatory therapy for distinct populations. Therefore, we aimed to establish an immune model to classify sepsis into different immune endotypes via transcriptomics data analysis of previously published cohort studies.

**Methods:** Datasets from two observational cohort studies that included 585 consecutive sepsis patients admitted to two intensive care units were downloaded as a training cohort and an external validation cohort. We analyzed genome-wide gene expression profiles in blood from these patients by using machine learning and bioinformatics.

**Results:** The training cohort and the validation cohort had 479 and 106 patients, respectively. Principal component analysis indicated that two immune subphenotypes associated with sepsis, designated the immunoparalysis endotype, and immunocompetent endotype, could be distinguished clearly. In the training cohort, a higher cumulative 28-day mortality was found in patients classified as having the immunoparalysis endotype, and the hazard ratio was 2.32 (95% CI: 1.53–3.46 vs. the immunocompetent endotype). External validation further demonstrated that the present model could categorize sepsis into the immunoparalysis and immunocompetent type precisely and efficiently. The percentages of 4 types of immune cells (M0 macrophages, M2 macrophages, naïve B cells, and naïve CD4 T cells) were significantly associated with 28-day cumulative mortality (*P* < 0.05).

**Conclusion:** The present study developed a comprehensive tool to identify the immunoparalysis endotype and immunocompetent status in hospitalized patients with sepsis and provides novel clues for further targeting of therapeutic approaches.

## Background

Sepsis is a highly heterogeneous syndrome associated with diverse immune status upon pathogen invasion. Normal immune responses can eradicate pathogens, and the pathophysiology of sepsis is caused by the inappropriate regulation of these normal reactions (1, 2). The extent of hyperactivated and hypoactivated immune responses vary among individuals, which results in heterogeneities in immune responses in sepsis (3, 4). It is urgent to clarify the immune status of sepsis to help identify patients who would benefit from immunomodulatory therapies (5–9).

Previous studies attempted to identify diverse immune statuses through clinical features or biomarkers. For example, Seymour et al. classified sepsis patients into four derived phenotypes based on 29 clinical features (temperature, mean arterial pressure, fluid resuscitation response, central venous oxygen saturation, etc.) (10). Using transcriptomic data, researchers identified four subphenotypes of sepsis; among them, one phenotype was associated with higher mortality than the other three phenotypes, which were associated with moderate mortality (11). However, the above described studies of phenotypes were qualitative rather than quantitative, and the immune state level was barely recognized. In addition, the use of one or two biomarkers, such as human leukocyte antigen-DR isotype (HLA-DR) and cytotoxic T lymphocyte-associated antigen-4 (CTLA-4), could not truly represent the global immune status. Moreover, false positive and false negative results might occur for various kinds of patients. Last but not least, routine parameters and biomarkers reflect surface-level phenomena associated with immune cell dysfunction and imbalance and are insufficiently robust to permit an actual intrinsic monitoring of immune status (12–15).

Recently, Newman et al. developed an algorithm to calculate the proportions of 22 types of human immune cells according to the ribonucleic acid (RNA) matrix (16) (using RNAomics or RNA-seq), and the proportions of these 22 human immune cell types have been confirmed to represent the immune status of human beings. Furthermore, it has been demonstrated that the CIBERSORT algorithm has higher accuracy and sensitivity than conventional technologies such as immunohistochemistry and flow cytometry (17, 18). To date, this algorithm has been widely utilized in assessing the immune status of patients with cancer for guiding immunotherapy, but it has never been used in sepsis patients. Thus, with the CIBERSORT approach, we assessed the proportions of 22 types of infiltrating immune cells based on two published cohort studies of sepsis. To analyze and quantitatively measure the patient immune responses to pathogens, an immune model for categorizing the immune endotypes of sepsis was constructed, and the immune cell subsets associated with potential therapeutic targets with prognostic value were also explored simultaneously.

## Methods

### Data Sources and Study Selection

A public database (GEO database) was searched for all expression microarrays that matched terms associated with sepsis. The datasets were collected from clinical studies investigating sepsis in adults using peripheral blood within 48 h after ICU admission. The exclusion criteria were as follows: (1) datasets that utilized endotoxin or lipopolysaccharide infusion like those used in *in vitro* or animal models of sepsis; (2) clinical gene expression microarray analyses derived from sorted cells; and (3) a sample size <100.

### Data Preprocessing

All datasets were downloaded as.txt files, and the outputs from the mRNA array were normal-exponential background-corrected and then between-array quantile-normalized using the limma R package. To ensure compatibility with the microarray study, expression was normalized using weighted linear regression, and the estimated precision weights of each observation were multiplied by the corresponding log2 value to yield the final gene expression values.

The dataset with the most complete prognostic data and the maximum sample size was used as the training cohort, and another dataset was used as the external validation cohort.

### Cell Type Identification by Estimating the Relative Subset of Known RNA Transcripts (CIBERSORT)

We used the CIBERSORT algorithm for quantification and discrimination of the absolute proportions of 22 human immune cell phenotypes from transcriptomic data, including seven T cell types (CD8 T cells, CD4 naïve T cells, CD4 memory resting T cells, CD4 memory activated T cells, follicular helper T cells, regulatory T cells, and gamma delta T cells), naïve and memory B cells, plasma cells, NK cells, and myeloid subsets. Immune cells are classified as high, median, and low expression according to the high and low interquartile ranges (IQRs). Pearson correlation analyses for various immune cell types were performed to assess the collinearity of the enrolled immune cells.

### Identification of Immune Cells With Prognostic Value and Construction of an Immunity Risk Model

The univariate Cox proportional hazards model with Bonferroni correction for multiple comparisons was used to determine the prognostic signatures with a cut-off value of *P* < 0.05 by using the survival R package. Then, both backward and forward stepwise selection with the Akaike information criterion (AIC) were used to identify the final variables for the multivariable Cox proportional hazards regression models through the survival R package.

The associations of relevant immune cell types with survival were assessed using multivariable Cox proportional hazard regression models. Hazard ratios (HRs) were presented with the 95% CIs. Selected variables were incorporated into the risk model to predict the probability of 28-day mortality using the rms R package. The risk scores for each sample were calculated according to the risk model. The respective medians of two clusters were used as the cut-off values to classify the patients as having either the Immunity-A endotype or the Immunity-B endotype.

### Assessment and Validation of the Immune Model

To multidimensionally evaluate the discrimination ability of the risk model in categorizing sepsis-induced immune dysfunction, we investigated the variation in immune cells, immune molecules, and immunity-related signal transduction pathways between the immunity-A endotype and immunity-B endotype. An empirical Bayesian approach was implemented to estimate immune cell and immune molecule changes using moderated *t*-tests. Gene set enrichment analysis (GSEA) was performed to assess immunity-related pathway activity variation between the Immunity-A and Immunity-B types. A *P* < 0.05 was set as the significance criterion. Kaplan-Meier (KM) curves and principal component analysis (PCA) were performed to evaluate the calibration capability of the risk model. External datasets were utilized for model validation. Perl 64 was used to merge data. Data processing, analysis, and diagram plotting were conducted in R x64 3.6.1.

### Sensitive Analysis

To further evaluate whether the current model could identify the immune status of a pneumonia and non-pneumonia induced sepsis population, the sensitive analyses were conducted to investigate discrimination ability of the current model in pneumonia and non- pneumonia patients respectively.

## Results

### Characteristics of the Datasets and Patients

After the search strategy and inclusion criteria were determined, 2 mRNA datasets from patients with sepsis (GSE65682 and GSE63042) were used to build the mRNA expression profiling datasets. The flow-process diagrams of the process of dataset screening are shown in Supplementary Figure 1. The GSE65682 dataset (479 patients with sepsis) was used as the training cohort since the contributors (University Medical Center in Utrecht and the Academic Medical Center in Amsterdam) uploaded relatively complete prognostic data, and this dataset had the maximum sample size. Simultaneously, GSE63042 (106 patients with sepsis) was used as the external validation cohort. All patients were older than 18 years and were diagnosed with sepsis. The septic shock ratios for GSE65682 and GSE63042 were 34.8 and 31.1%, respectively. Details of the demographic and clinical characteristics are shown in Table 1.

**Table 1:** Demographic and clinical characteristics.

	**GSE65682 (*N* = 479)**	**GSE63042 (*N* = 106)**
Male sex	272 (56.8%)	63 (59.4%)
Age	63 (18–89)	59 (38–85)
Country	Netherlands	USA
Pneumonia diagnoses	183 (38.0%)	24 (22.6%)
Septic shock	167 (34.8%)	33 (31.1%)
28 day mortality	115 (24.0%)	28 (26.4%)
Main study	Classification for sepsis through transcriptomic data	Bioinformatic analysis for host response in sepsis

*N, number*.

### Construction of the Immunity Risk Model

**Figure 1:**
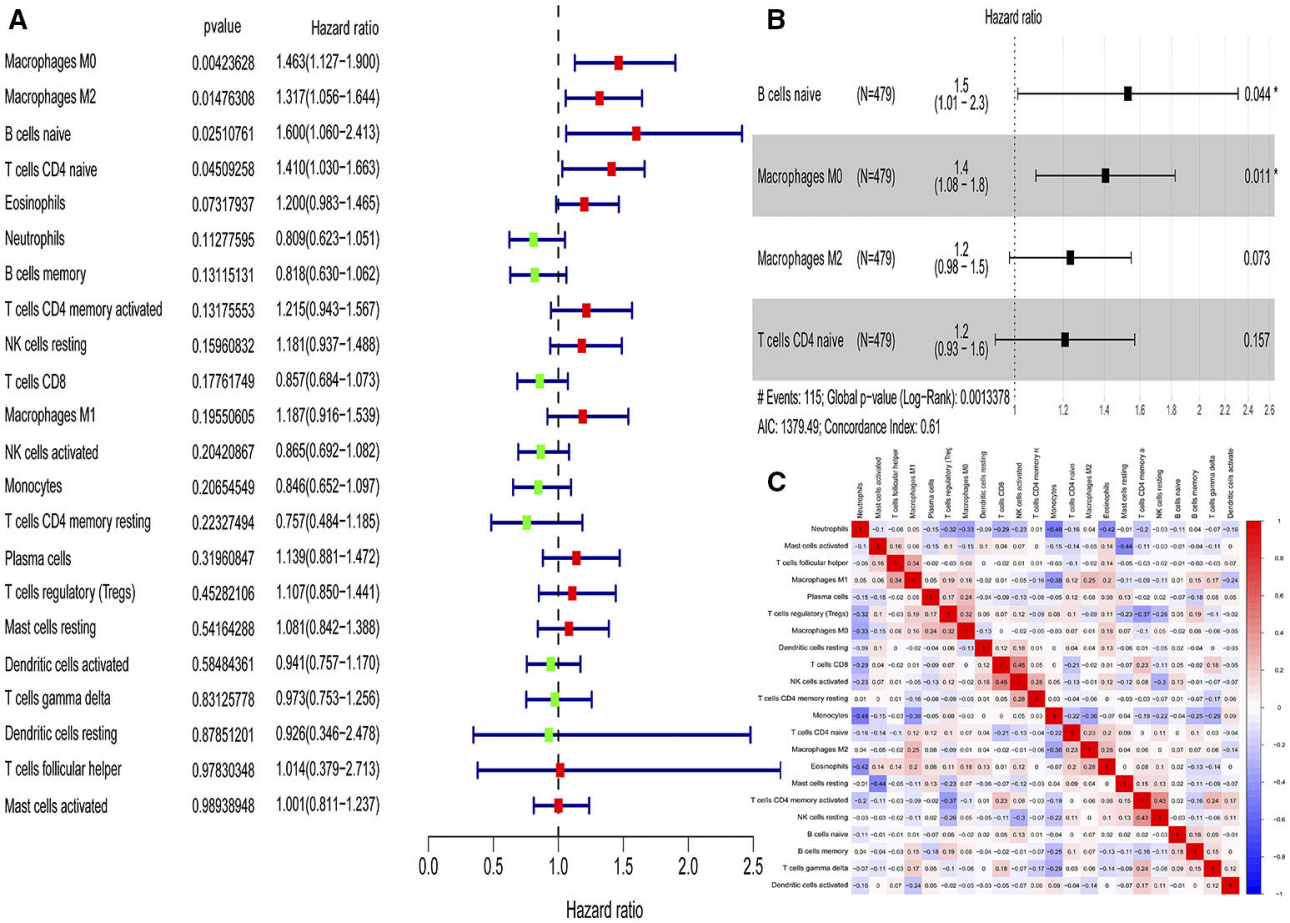
Identification of immune cells with prognostic value and construction of an immunity risk model. **(A)** Forest plots of univariate Cox proportional hazard analysis for the identification of prognostic immune cells (red forest plots represent hazard factors, and green forest plots represent protective factors). **(B)** Forest plots of multivariable Cox proportional hazard analysis for the construction of the immunity risk model (AIC, Akaike information criterion), **(C)** Correlation heat map for the assessment of collinearity (numbers in the heatmap represent Pearson correlation coefficients; red represents a positive correlation and blue represents a negative correlation). *P < 0.05.

According to the univariate Cox regression analyses and stepwise selection, the percentages of 4 immune cell types (M0 macrophages, M2 macrophages, naïve B cells, and naïve CD4 T cells) were significantly associated with 28-day cumulative mortality (Figure 1A). The 4 identified immune cell types were included in the immunity risk model generated through multivariate Cox regression (Figure 1B). Each patient was assigned a risk score through this model. Correlation analyses among various immune cell types to find the links among immune cells was shown in Figure 1C.

### Model Assessment

**Figure 2:**
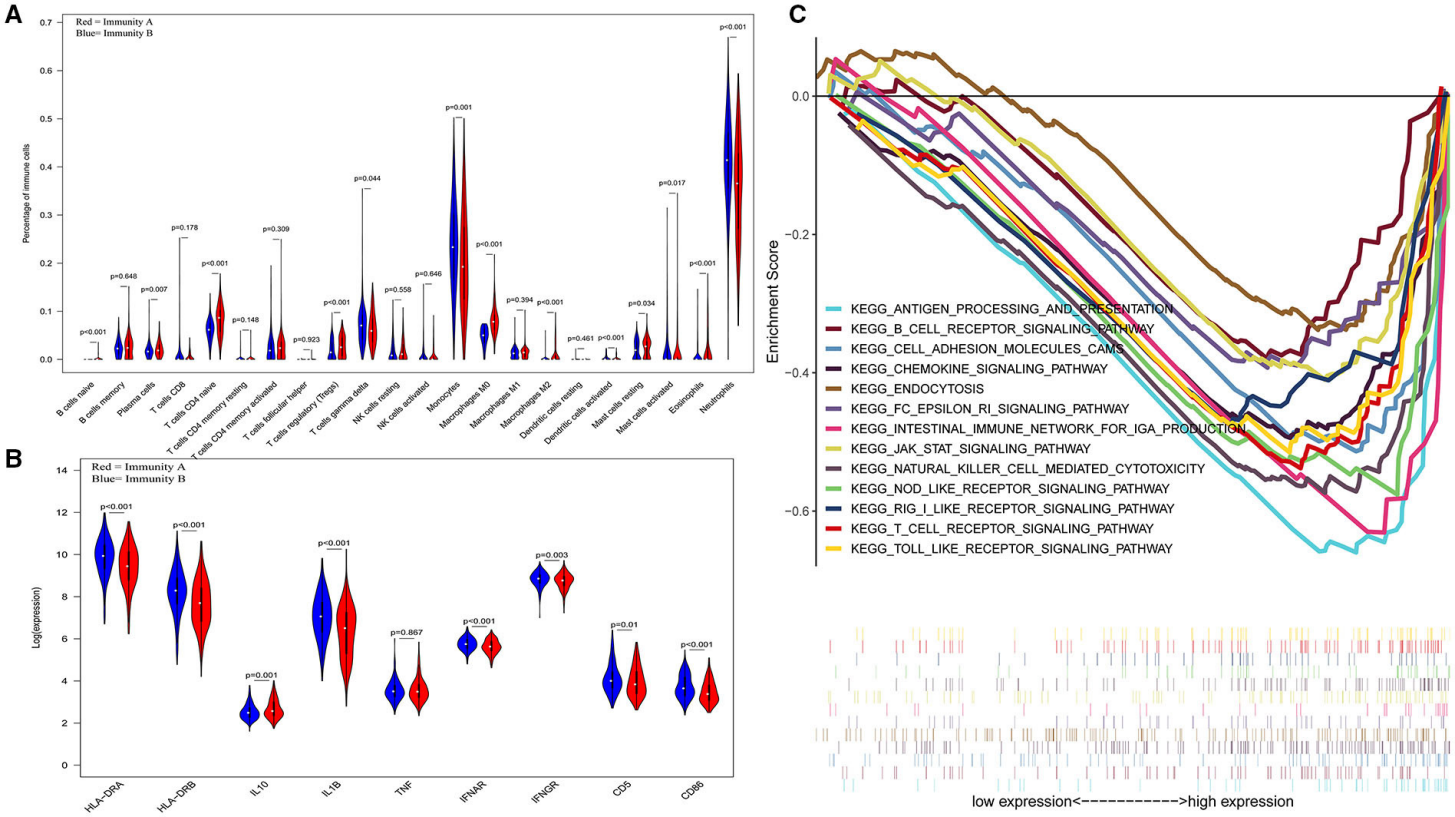
Three-dimensional assessments demonstrate that patients with the immunity-A endotype suffer from immune suppression. **(A)** Violin plots of immune cell analyses (medians, quartiles, extremums, data distributions, and *P*-values for the difference analysis are described in the violin plots. The percentages of immune-enhancing cells (neutrophils, gamma delta T cells, activated dendritic cells, and activated mast cells) were significantly decreased in the immunity-A endotype. The percentages of immunosuppressive cells (regulatory T cells, M2 macrophages) and naïve immune cells (naïve B cells, naïve CD4 T cells and M0 macrophages)—were obviously upregulated in the immunity-A endotype. **(B)** Violin plots of the immune molecule difference analysis. The immune-enhancing molecules (HLA-DRA, HLA-DRB, IL1B, IFNAR, IFNGR, CD5, and CD86) were significantly downregulated and an immunosuppressive molecule—IL10—was obviously upregulated in the immunity-A endotype. **(C)** Diagrams of the Gene Set Enrichment Analysis. The innate immunity, humoral immunity, cellular immunity, and promotion of immunity-related pathways were all suppressed in the immunity-A endotype group.

The three-dimensional results (immune cells, immune molecules, and immunity-related pathways) demonstrate that this risk model could stratify sepsis patients with either immunocompetent status or immunoparalysis. Patients with the immunity-B endotype displayed an immunocompetent status, while the immunity-A endotype patients suffered from immunoparalysis (Figure 2). At the level of immune cells, differential expression analysis indicated that the percentages of immune-enhancing cells (neutrophils, gamma delta T cells, activated dendritic cells, and activated mast cells) were significantly downregulated in the immunity-A endotype (Figure 2A) compared with those in the immunity-B endotype, *P* < 0.05. Moreover, the percentages of immunosuppressive cells (regulatory T cells and M2 macrophages) and naïve immune cells (naïve B cells, naïve CD4 T cells, and M0 macrophages) were obviously upregulated in the immunity-A endotype compared with those in the immunity-B endotype, *P* < 0.05.

On the other hand, immune-enhancing molecules (HLA-DRA, HLA-DRB, IL1B, IFNAR, IFNGR, CD5, and CD86) were significantly downregulated, and immunosuppressive molecules (IL10) were obviously upregulated in the immunity-A endotype compared with those in the immunity-B endotype at the molecular level according to the violin plot (Figure 2B), *P* < 0.05.

Finally, at the level of immunity-related signal transduction pathways, GSEA demonstrated that immune enhancement-related pathways were significantly suppressed in the immunity-A endotype in sepsis (Figure 2C). In contrast, these pathways were activated in the immunity-B endotype. The summary view of the GSEA results in the training cohort is shown in Figure 2C; the details for every pathway are shown in Supplementary Figures 2–5. These pathways could be classified as associated with innate immunity (endocytosis and natural killer cell-mediated cytotoxicity), humoral immunity (antigen processing and presentation, B cell receptor signaling pathway, and intestinal immune network for IgA production), cellular immunity (T cell receptor signaling pathway and Toll-like receptor signaling pathway), and the promotion of immunity (Fc epsilon RI signaling pathway, chemokine signaling pathway, RIG-I-like receptor signaling pathway, and NOD-like receptor signaling pathway).

**Figure 3:**
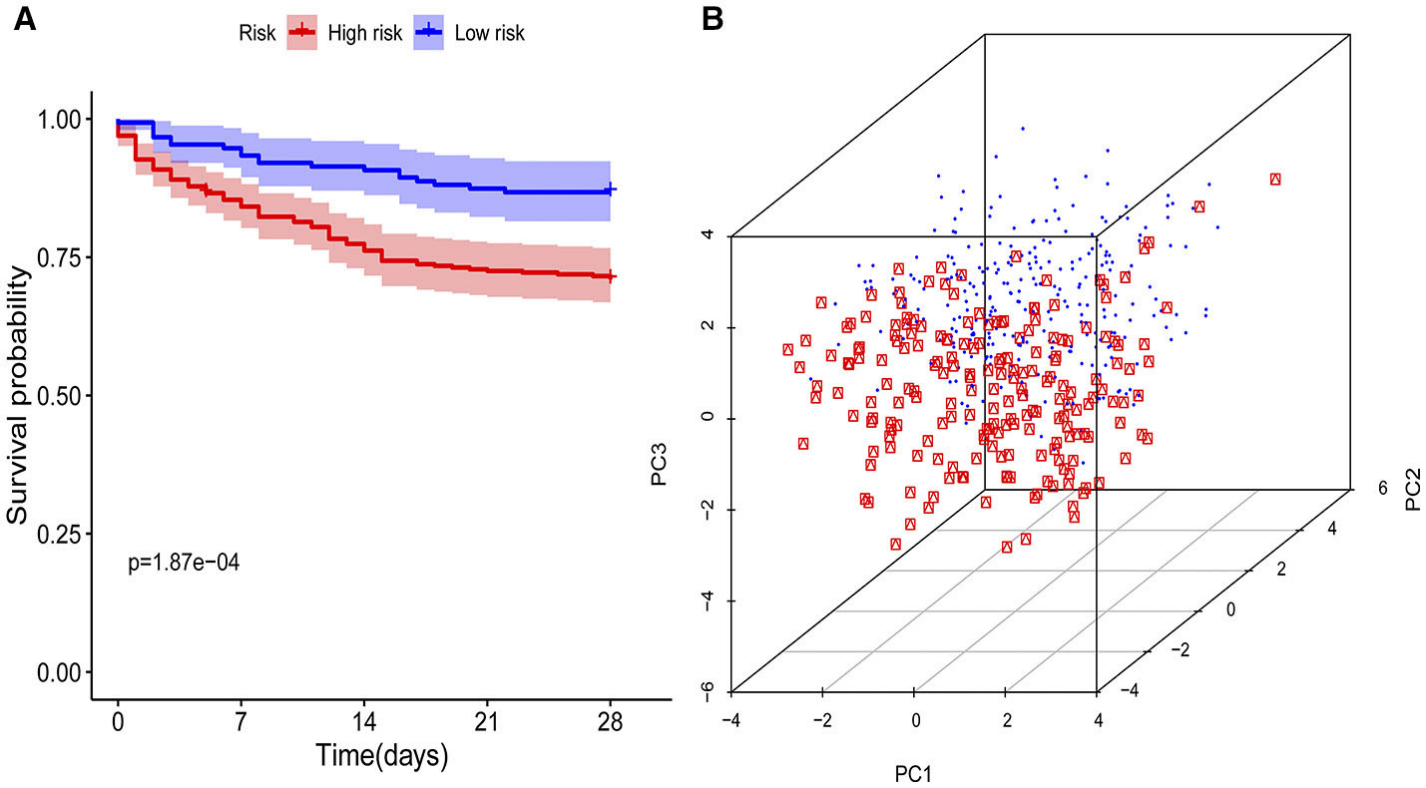
Survival curves and principal component analysis for evaluating the calibration ability of the immunity risk model. **(A)** Survival curves of the immunity-A and immunity-B endotypes (Kaplan-Meier curves indicate that the immunity-A endotype was associated with significantly higher mortality than the immunity-B endotype), **(B)** Principal component analysis between the immunity-A and immunity-B endotypes (the red dots represent patients with the immunity-A endotype, and the blue dots represent patients with the immunity-B endotype; an obvious clustering trend can be found).

The KM curves indicated that the immunity-A endotype was associated with a significantly higher cumulative 28-day mortality rate compared to the immunity-B endotype, with a hazard ratio (95% CI) of 2.32 (1.53–3.46) and a *P*-value of 0.00 (Figure 3A). PCA shows an obvious clustering trend for immune status between the Immunity-A and Immunity-B endotypes (Figure 3B).

### Sensitivity Analysis

**Figure 4:**
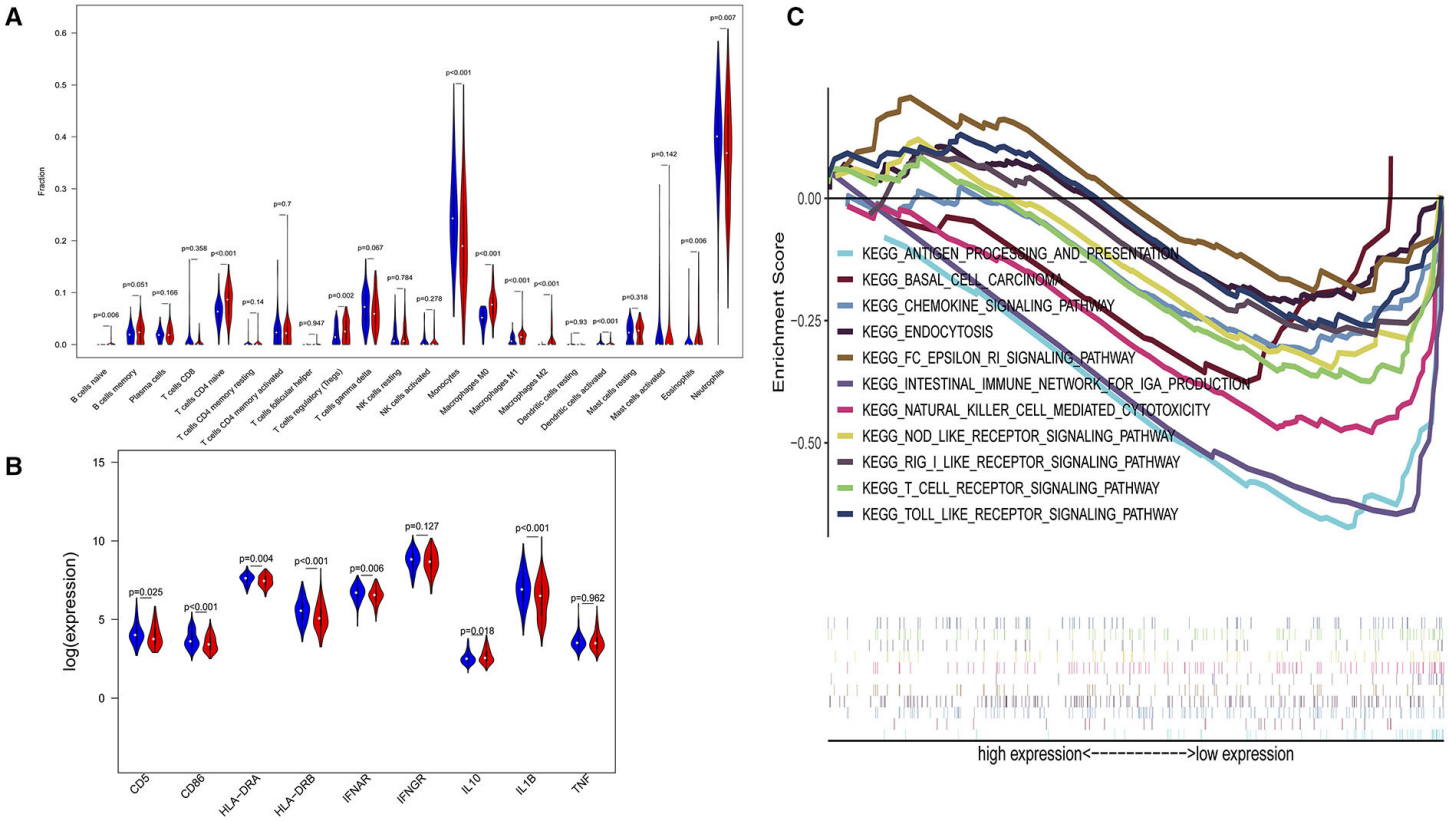
Subgroup analysis on septic population after removing patients with pneumonia. **(A)** Violin plots of immune cell difference analyses (medians, quartiles, extremums, data distributions, and *P*-values for the difference analysis are described in violin plots. The percentages of immune-enhancing cells (activated neutrophils and dendritic cells) were significantly downregulated in the immunity-A endotype. The percentages of immune suppressive cells (regulatory T cells, and macrophage M2) and naïve immune cells (naïve B cells, naïve CD4 T cells, and M0 macrophages) were obviously upregulated in the immunity-A endotype). **(B)** Violin plots of the immune molecule difference analysis (immune-enhancing molecules—HLA-DRA, HLA-DRB, IL1B, IFNAR, CD5, and CD86—were significantly downregulated and the immunosuppressive molecule IL10 was obviously upregulated in the immunity-A endotype). **(C)** Diagrams of the Gene Set Enrichment Analysis. The innate immunity, humoral immunity, cellular immunity, and promotion of immunity-related pathways were all suppressed in the immunity-A endotype group.

**Figure 5:**
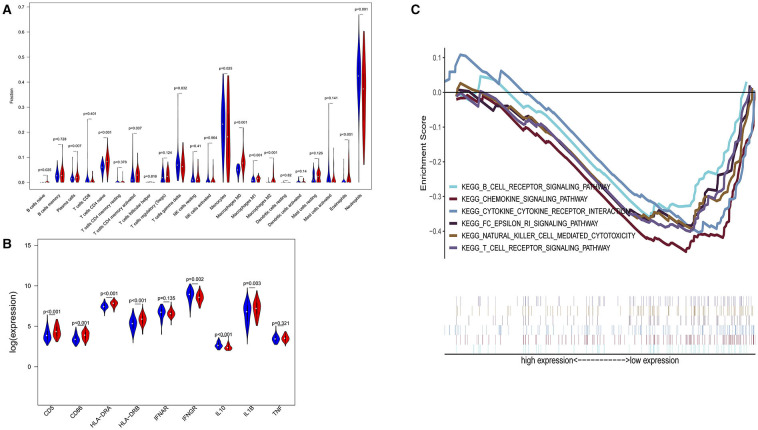
Subgroup analysis on septic population with pneumonia. **(A)** Violin plots of immune cell difference analyses (medians, quartiles, extremums, data distributions, and *P*-values for the difference analysis are described in violin plots. The percentages of immune-enhancing cells—activated neutrophils- were significantly downregulated in the immunity-A endotype. The percentages of immune suppressive cells—M2 macrophages—and naïve immune cells (naïve B cells, naïve CD4 T cells, and M0 macrophages) were obviously upregulated in the immunity-A endotype). **(B)** Violin plots of the immune molecule difference analysis. The immune-enhancing molecules—HLA-DRA, IL1B, IFNGR, CD5, and CD86—were significantly downregulated and the immunosuppressive molecule IL10 was obviously upregulated in the immunity-A endotype. **(C)** Diagrams of the Gene Set Enrichment Analysis. The innate immunity, humoral immunity, cellular immunity, and promotion of immunity-related pathways were all suppressed in the immunity-A endotype group.

In a sensitivity analysis evaluating the removal of sepsis induced by pneumonia in GSE65682, similar results in the overall population are observed which are shown in Figures 4, 5. However, this sensitivity analysis could not be done in GSE63042, since the original case data of individuals were not provided by researchers.

### External Validation

To validate the model of Immunity-A and Immunity-B, the GSE63042 datasets were set as the external validation cohort. External validation further confirms that the ability of this model to categorize based on immune dysfunction is efficient and precise.

**Figure 6:**
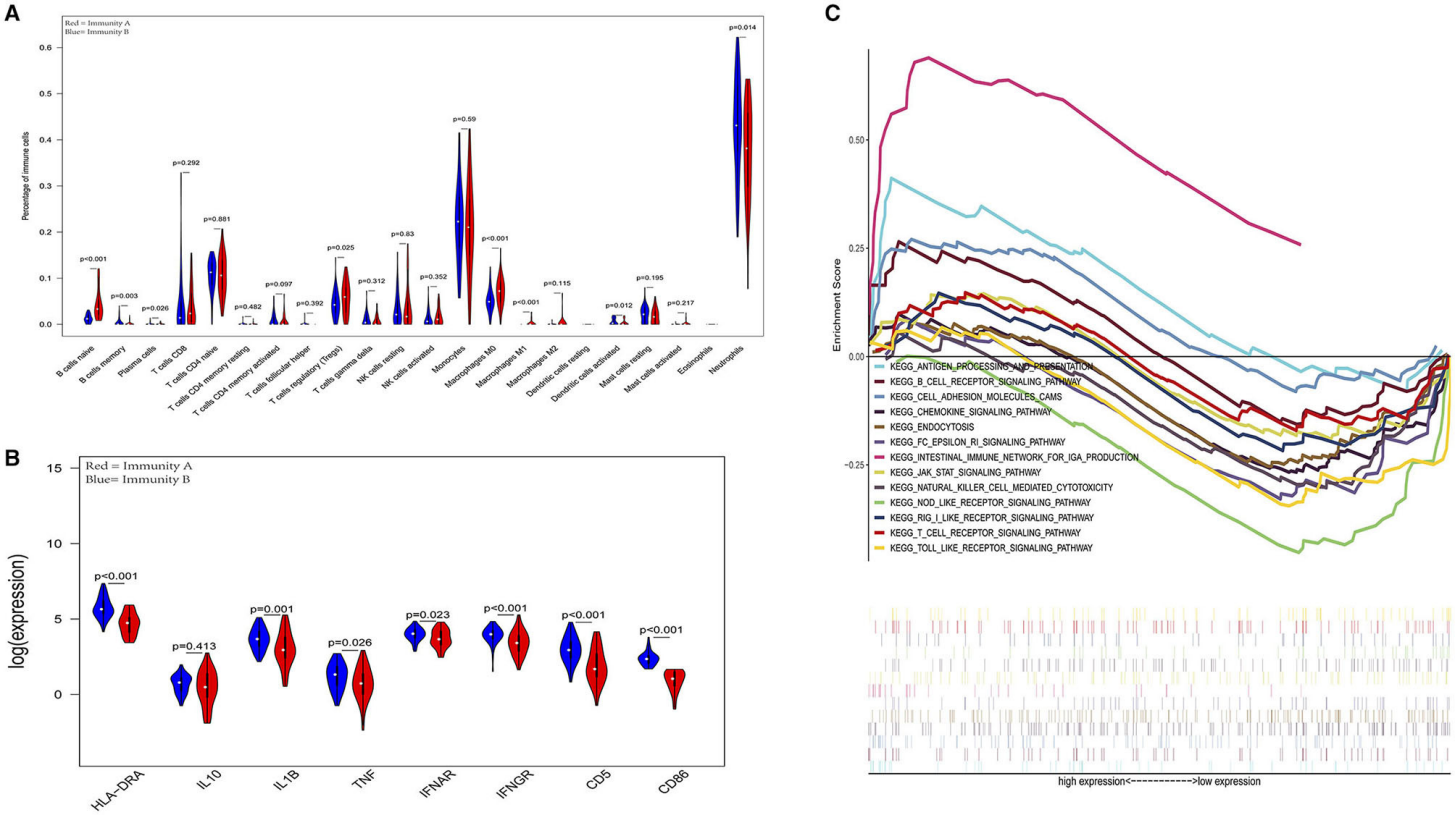
External validation further demonstrates that patients in the immunity-A endotype suffer from immune suppression. **(A)** Violin plots of immune cell difference analyses (medians, quartiles, extremums, data distributions, and *P*-values for the difference analysis are described in violin plots. The percentages of immune-enhancing cells (activated neutrophils and dendritic cells) were significantly downregulated in the immunity-A endotype. The percentages of immune suppressive cells (regulatory T cells) and naïve immune cells (naïve B cells, naïve CD4 T cells, and M0 macrophages) were obviously upregulated in the immunity-A endotype). **(B)** Violin plots of the immune molecule difference analysis. The immune-enhancing molecules (HLA-DRA, IL1B, IFNAR, IFNGR, CD5, and CD86) were significantly downregulated and the immunosuppressive molecule IL10 was obviously upregulated in the immunity-A endotype. **(C)** Diagrams of the Gene Set Enrichment Analysis (most immune enhancement-related pathways except humoral immunity-related pathways are significantly suppressed in the immunity-A endotype).

In the external validation cohort, analysis of the levels of immune cells, immune molecules, and immune pathways robustly confirmed that patients in the Immunity-A endotype classified by the current model suffered from immunoparalysis. Conversely, patients in the immunity-B endotype showed immunocompetent status (*P* < 0.05) (Figure 6). The details of every pathway are shown in Supplementary Figures 6–9.

## Discussion

Accumulating evidence supports the central role of the immune system in the pathogenesis of sepsis, a better insight to uncover the immunological phenotype of sepsis patients is crucial for effective immunomodulatory treatment. The current study is the first to identify two distinct immune endotypes based on the microarray data of sepsis patients by using CIBERSORT analysis and provides novel evidence and clues for further research on the molecular mechanisms of sepsis. In particular, sepsis can be divided into subphenotypes based on infiltrating immune cell characteristics. The immunocompetent subphenotype (immunity-B endotype) is characterized by increased expression levels of immune response-associated molecules, decreases in immature immune cells (naïve B cells, naïve CD4 T cells, and M0 macrophages), and increased activity of immune-enhancing pathways compared to the immunity-A endotype (immunoparalysis). In addition, we also revealed that elevations in M0 macrophages, M2 macrophages, naïve B cells, and naïve CD4 T cells in peripheral blood were independent risk factors for poor prognosis in sepsis at onset. Patients with the immunity-A endotype were confirmed as having immunoparalysis and a higher cumulative 28-day mortality, and patients with the immunity-B endotype seemed to have an immunocompetent status and a higher survival rate. The immune score calculated by this model could represent the severity of immunoparalysis.

Normal immune and physiologic responses eradicate pathogens, and the pathophysiology of sepsis is due to the improper regulation of these normal reactions. Pathogen contact with the inflammatory system should eliminate the microbe and rapidly return the host to homeostasis. The septic response may accelerate due to continued activation of macrophages/monocytes, which play a key role in the regulation of both innate and adaptive immunity. The large contribution to immune suppression of peripheral blood mononuclear cells (including macrophages and T and B lymphocytes) reveal the downregulation of genes involved in the inflammatory response and the increased expression of genes involved in apoptosis. Massive mononuclear cell death leads to naïve cell proliferation in the bone marrow. These findings may explain why immature peripheral blood mononuclear cells were more common in the immune A endotype.

A number of alterations in the expression of distinct cell surface markers, such as HLA-DRA, HLA-DRB, IL1B, IFNAR, IFNGR, CD5, and CD86, have been described in these two endotypes, and these molecules were defined as immunoactivated molecules in previous studies (19, 20). Furthermore, Venet et al. and Carson et al. showed that sepsis induced an increase in the proportion of anti-inflammatory immune cells (such as Tregs) that release anti-inflammatory cytokines (such as IL10), which resulted in epigenetic alterations of naïve immune cells and further suppressed inflammatory activation-related pathways (such as the Toll-like receptor signaling pathway) (21, 22). To date, researchers believe that immunoparalysis is an independent risk factor for poor prognosis in sepsis (19, 20), which was also confirmed in our study. Therefore, it was indicated that an increase in the proportion of naïve immune cells and immunosuppressive cells were the essential characteristics of immunoparalysis in sepsis.

However, previous studies of immunoparalysis in sepsis evaluated only some immune features (single immune cells or immune molecules) and lacked global assessment and validation (19–22). Therefore, the present study attempted to explore immune models appropriate for identifying immunoparalysis in sepsis via multiple parameters. Robustly, the discrimination performance of the current model was confirmed according to the assessment of immune cells, immune molecules, immune signal transduction pathways, and survival curves. Differential expression analysis of immune cells demonstrated that patients with the immunity-A endotype suffered from immune paralysis due to decreases in immune-enhancing cells, increases in immunosuppressive cells and increases in naïve immune cells. Poll et al. pointed out that the characteristics of immune suppression in sepsis were the low expression of HLA-DR on blood leucocytes and the high expression of IL-10 (an anti-inflammatory molecule), which could also be found in the immunity-A endotype, as shown by the violin plot of immune molecules (23–25). Furthermore, GSEA suggested that innate immunity-, cellular immunity-, and humoral immunity-related biological pathways were all suppressed in the high-risk group (26–28). In addition, the KM curves obviously suggested that patients in the high group (immunoparalysis) had decreased survival and poor prognosis. The external validation cohorts further demonstrated that the current model could effectively identify patients with the immunity-A endotype (immunoparalysis).

Sepsis 3.0 is defined as a life-threatening condition of organ dysfunction caused by the dysregulation of the host immune response to infection. The most important question is whether therapeutic interventions that target specific immune process mechanisms implicated in the pathophysiological changes of sepsis might further improve the therapeutic effects. It was reported that the number of immunotherapy studies of sepsis is almost 1,000 to date, but none of the results have been used in clinical practice. The primary reason for this is the lack of recognition of patient immune status. In future RCTs, scholars could use this model to categorize sepsis to design more precise immune therapies. In addition, our model could help clinicians identify patients with immunoparalysis. Avoidance of superinfection and the use of immunity enhancement drugs (such as interferon or thymosin) should be considered in these patients. In contrast, corticosteroids could be safely used for patients with low immunity risk scores calculated by this model in consideration of the effects of corticosteroids on improving the cardiovascular response to exogenous catecholamines. Furthermore, the present study demonstrated that naïve immune cells (M0 macrophages, naïve B cells, and naïve T cells) and immunity-regulating cells (Tregs and M2 macrophages) were significantly increased in the poor prognostic group. These results were similar to those of previous studies showing that immunoparalysis is crucially detrimental to sepsis patient survival. Due to the fast development and wide applications of next-generation sequencing (NGS) technologies, genomic sequence information is within reach to aid in the achievement of goals to determine the immune status in patients with sepsis onset and improve the survival of sepsis patients. The alterations of these immune cells could be used as potential therapeutic targets to improve the treatment strategies for sepsis.

There are several limitations to the present study. First, as a retrospective study of primarily publicly available data, the demographics and clinical features such as severity, complications, and individual treatment of each patient for detailed could not be acquired. Thus, the sensitivity and longitudinal analyses cannot be totally completed. This may restrict the generalizability of the present model. Second, despite the use of two external validation cohorts, we do not present the results for any prospective clinical studies using this model. Prospective RCTs will be paramount in translating the results to clinical applications. In addition, despite a seemingly large sample size, we were unable to perform robust subgroup analyses (based on infection site or pathogen type) due to the lack of relevant information in public databases. In addition, this model was not sensitive enough to identify a hyperactivated immune response to sepsis because it was constructed based on naïve immune cells and M2 macrophages (screened by prognostic analysis). The patients with poor prognosis in this database mainly suffered from early immunosuppression (9, 11).

In conclusion, the present study developed a comprehensive tool to identify immunoparalysis endotypes and immunocompetent status in sepsis patients that have been hospitalized, and provides novel clues for further targeting of therapeutic approaches.

## Data Availability Statement

The original contributions presented in the study are included in the article/Supplementary Materials, further inquiries can be directed to the corresponding author/s.

## Ethics Statement

Written informed consent was not obtained from the individual(s) for the publication of any potentially identifiable images or data included in this article.

## Author Contributions

SZ had full access to all of the data in the study and took responsibility for the integrity and the accuracy of the data analysis. SZ, WC, and ZW performed the data download, bioinformatic analysis, and preparation of the article for publication. All authors participated in writing the article and preparing the figures.

## Conflict of Interest

The authors declare that the research was conducted in the absence of any commercial or financial relationships that could be construed as a potential conflict of interest.
