# Supplementary material for: Classification of Patients With Sepsis According to Immune Cell Characteristics: A Bioinformatic Analysis of Two Cohort Studies
Source: Front Med (Lausanne). 2020 Dec 3;7:598652. doi: 10.3389/fmed.2020.598652 (PMC7744969; doi:10.3389/fmed.2020.598652)
Supplement: Supplementary file 1 [file Data_Sheet_1.DOC]

Catalogue of Supplemental Material

| ***Title*** | ***Content*** | ***Page*** |
| --- | --- | --- |
| ***SM Figure 1*** | **The flow-process diagram for screening datasets.** | 2 |
| ***SM Figure 2*** | **GSEA for innate immunity related pathways in train cohort.** | 3 |
| ***SM Figure 3*** | **GSEA for humoral immunity related pathways. in train cohort.** | 4 |
| ***SM Figure 4*** | **GSEA for cellular immunity related pathways in train cohort.** | 5 |
| ***SM Figure 5*** | **GSEA for promoting immunity related pathways in train cohort.** | 6 |
| ***SM Figure 6*** | **GSEA for innate immunity related pathways in validation cohort.** | 7 |
| ***SM Figure 7*** | **GSEA for humoral immunity related pathways. in validation cohort.** | 8 |
| ***SM Figure 8*** | **GSEA for cellular immunity related pathways in validation cohort.** | 9 |
| ***SM Figure 9*** | **GSEA for promoting immunity related pathways in validation cohort.** | 10 |

SM= Supplemental Material


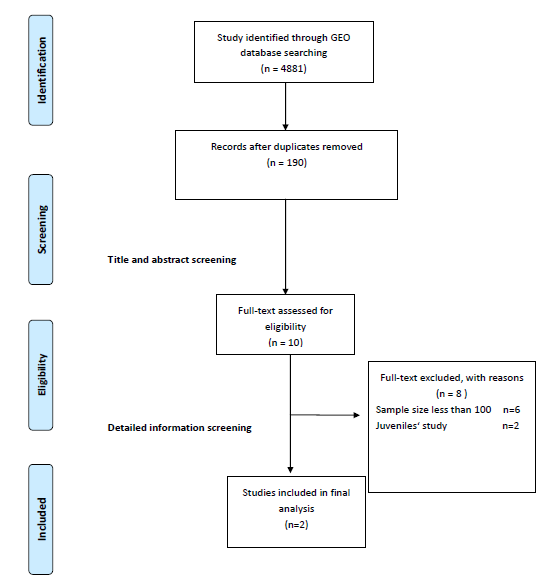


**SM Figure 1:The flow-process diagram for screening datasets.**


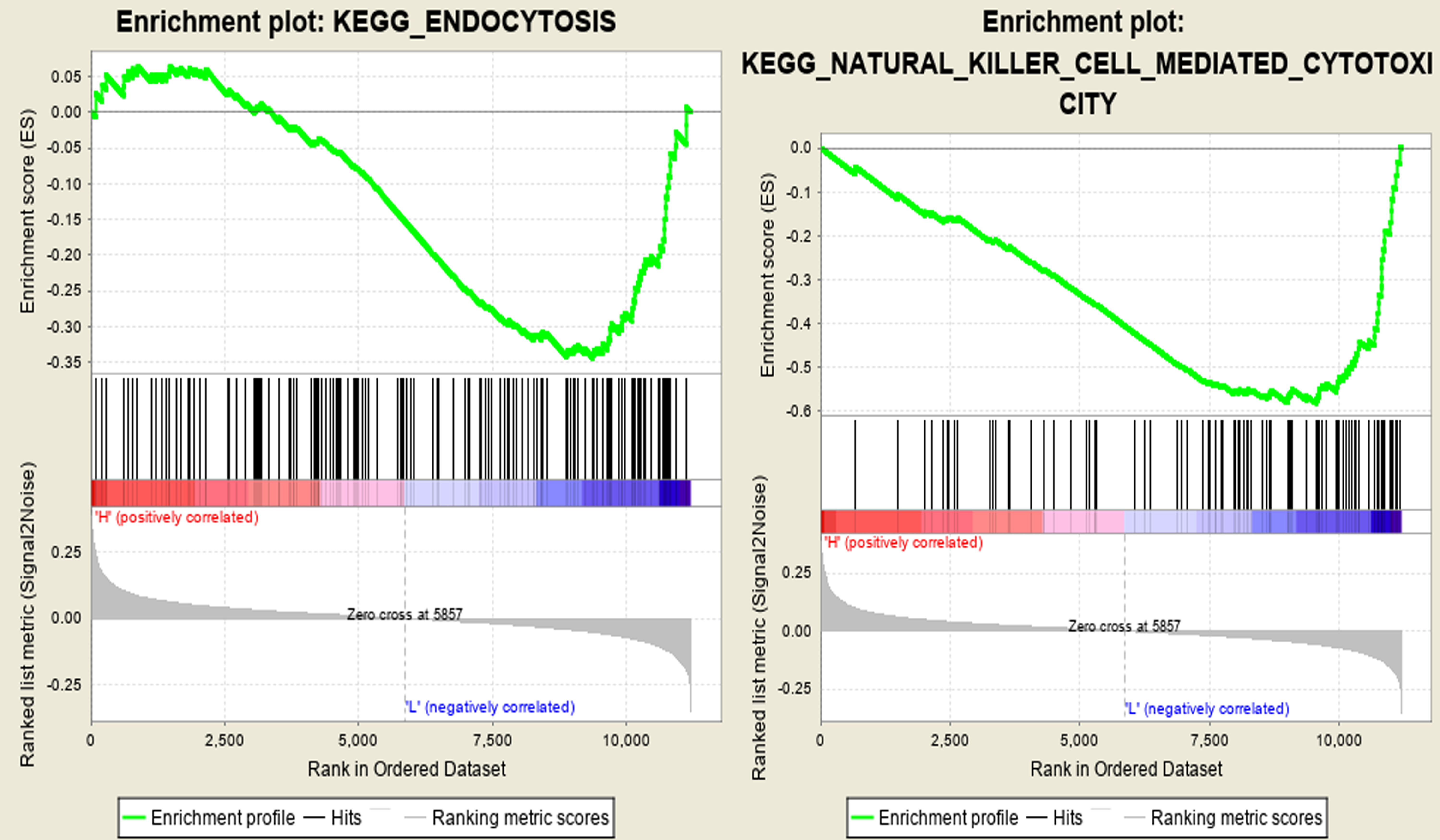


**SM Figure 2: GSEA for innate immunity related pathways in train cohort.**

The results indicated that endocytosis and natural killer cell mediated cytotoxicity are significantly suppressed in immunity-A endotype. GSEA: Gene Set Enrichment Analysis.


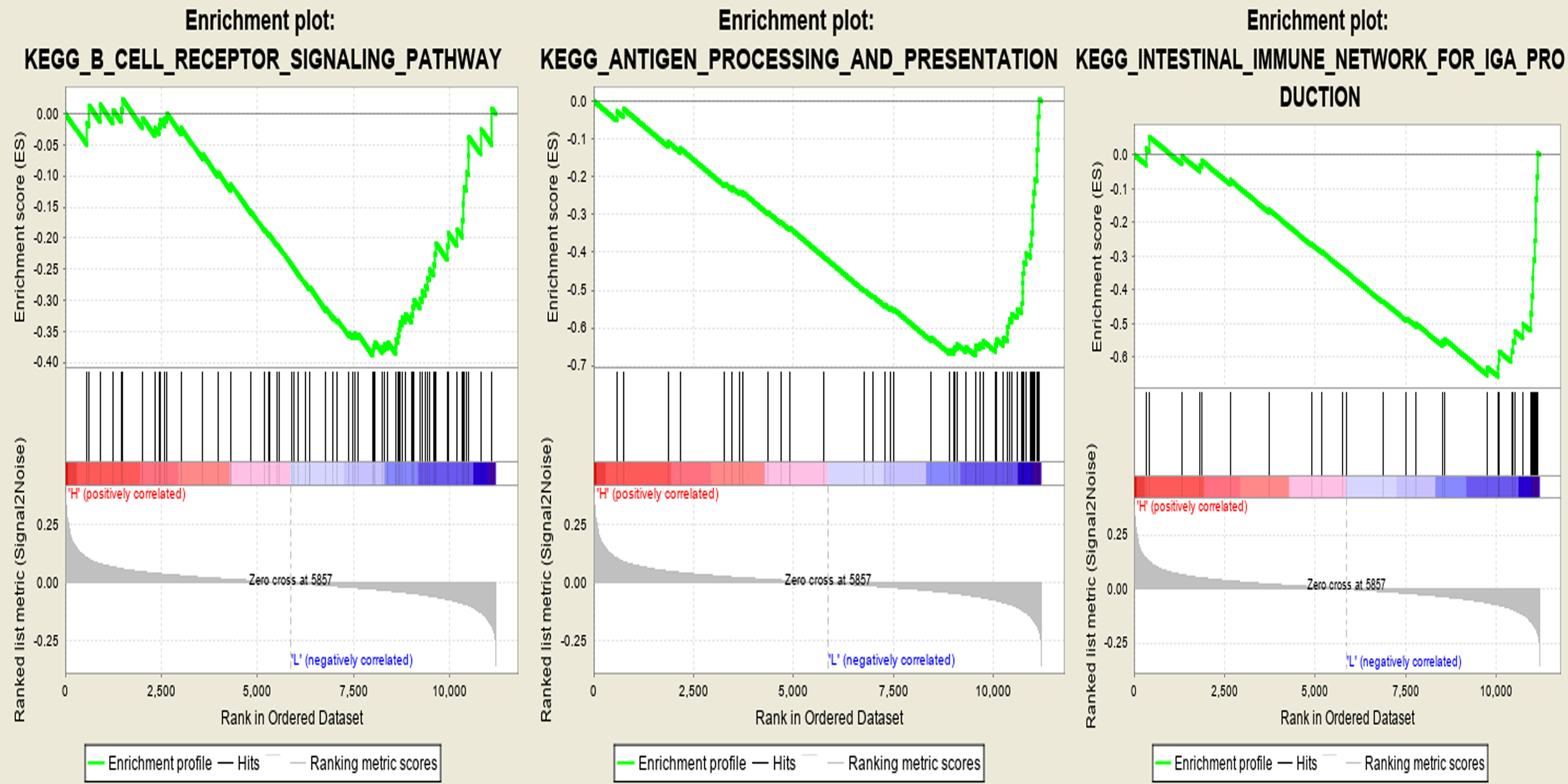


**SM Figure 3: GSEA for humoral immunity related pathways in train cohort.**

The results indicated that antigen processing and presentation, B cell receptor signaling pathway and intestinal immune network for IgA production are significantly suppressed immunity-A endotype. GSEA: Gene Set Enrichment Analysis.


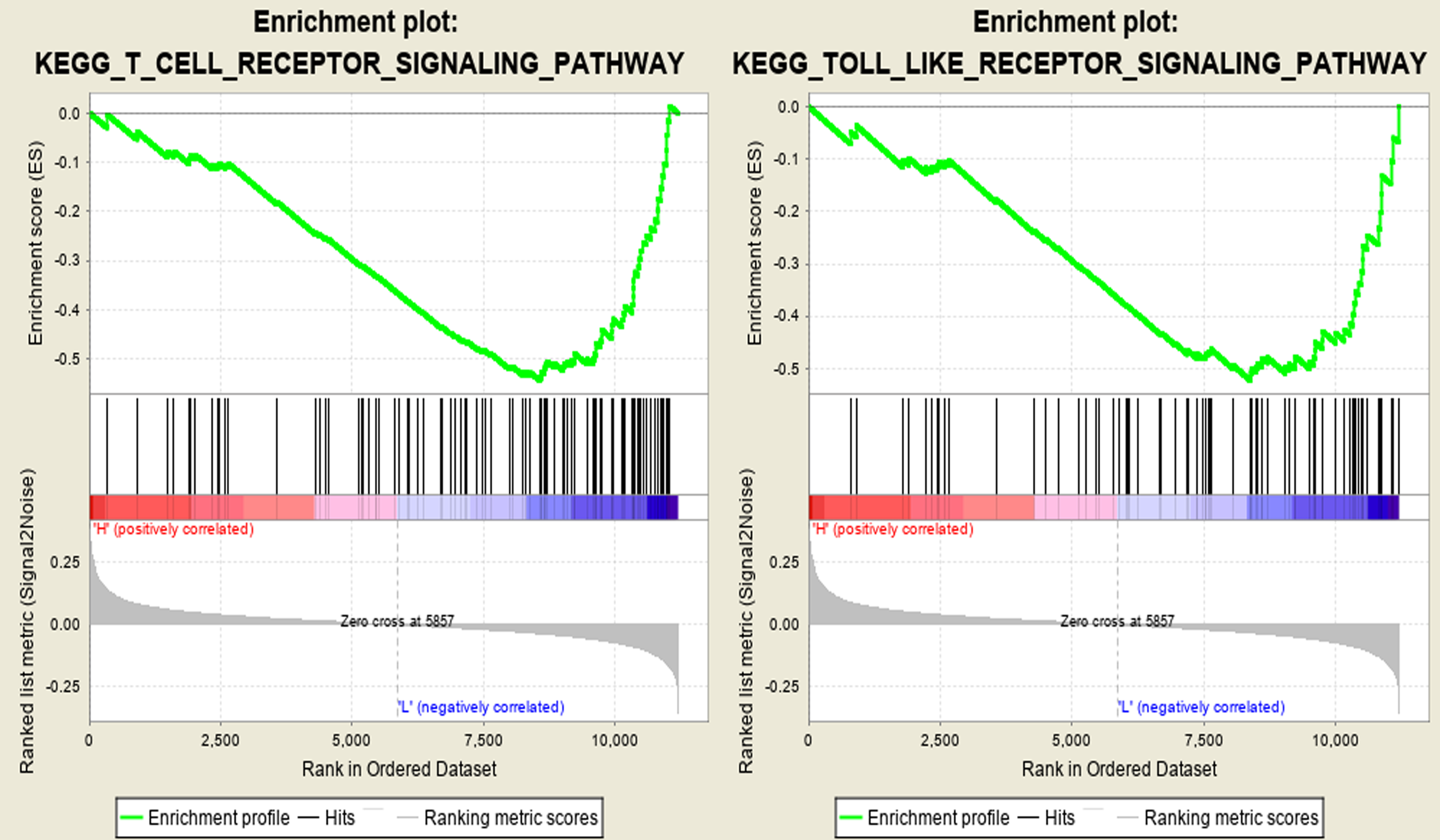


**SM Figure 4: GSEA for cellular immunity related pathways in train cohort.**

The results indicated that T cell receptor signaling pathway and toll like receptor signaling pathway for IgA production are significantly suppressed immunity-A endotype. GSEA: Gene Set Enrichment Analysis.

**
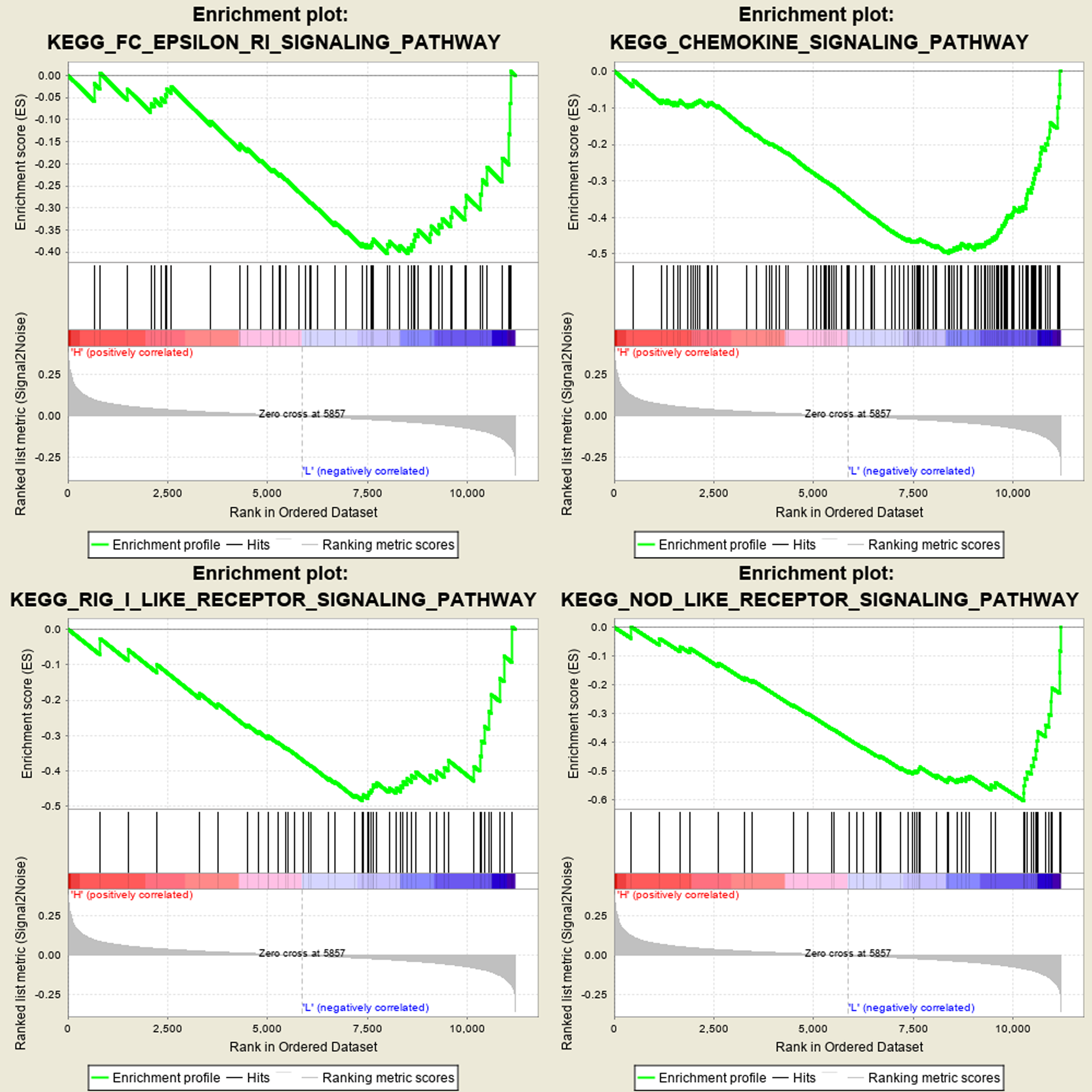
**

**SM Figure 5: GSEA for promoting immunity related pathways in train cohort.**

The results indicated that Fc epsilon RI signaling pathway, chemokine signaling pathway, RIG-I-like receptor signaling pathway and NOD like receptor signaling pathway are significantly suppressed immunity-A endotype. GSEA: Gene Set Enrichment Analysis.


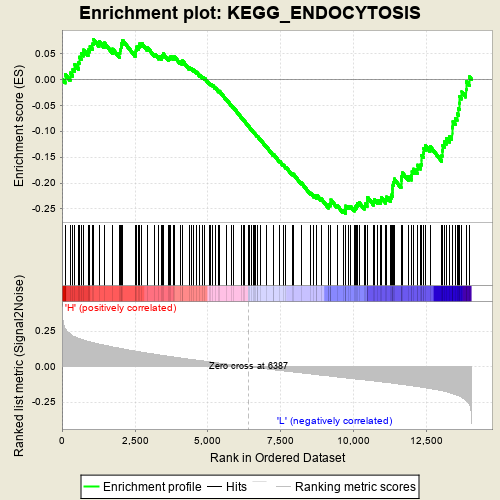

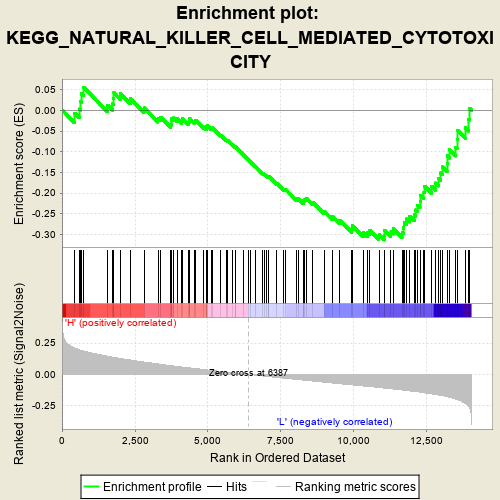


**SM Figure 6: GSEA for innate immunity related pathways in validation cohort.**

The results indicated that endocytosis and natural killer cell mediated cytotoxicity are significantly suppressed immunity-A endotype. GSEA: Gene Set Enrichment Analysis.


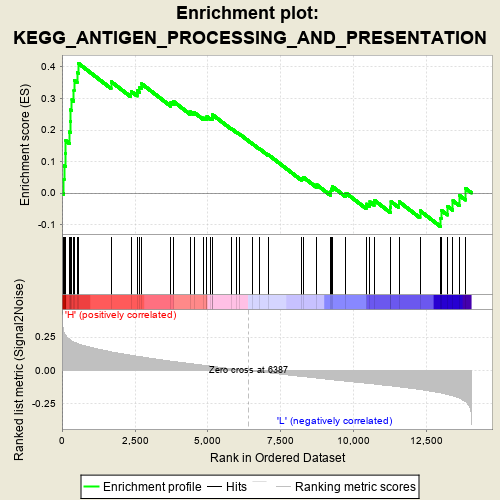

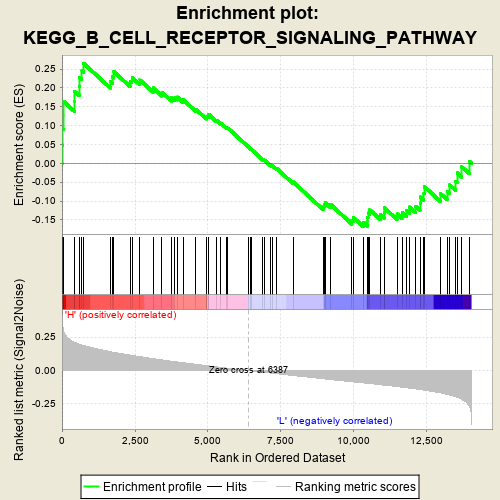

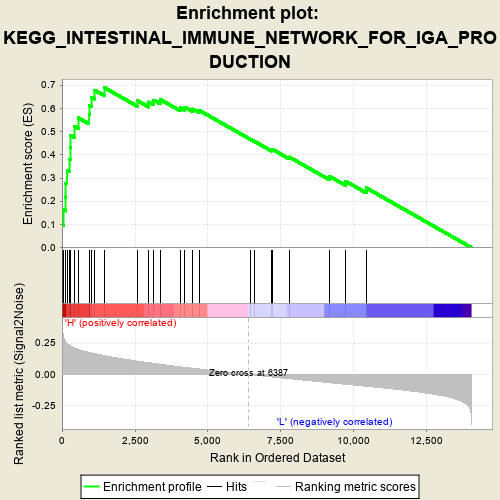


**SM Figure 7: GSEA for humoral immunity related pathways in validation cohort.**

The results indicated that antigen processing and presentation, B cell receptor signaling pathway and intestinal immune network for IgA production are not suppressed immunity-A endotype. GSEA: Gene Set Enrichment Analysis.


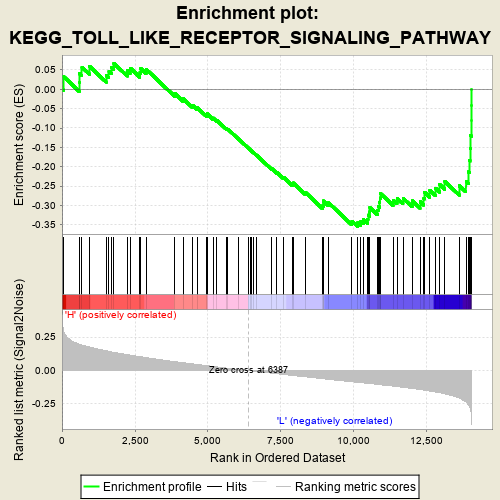

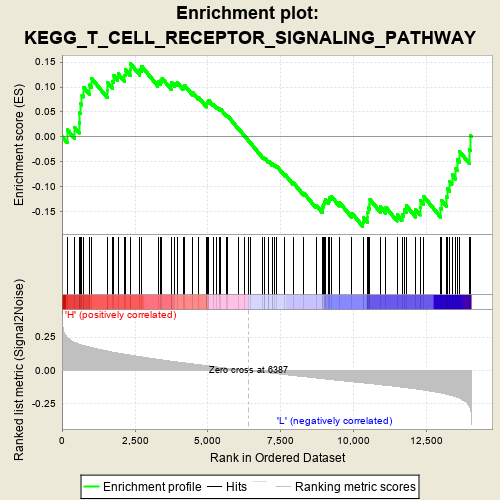


**SM Figure 8: GSEA for cellular immunity related pathways in validation cohort.**

The results indicated that T cell receptor signaling pathway and toll like receptor signaling pathway for IgA production are significantly suppressed immunity-A endotype. GSEA: Gene Set Enrichment Analysis.


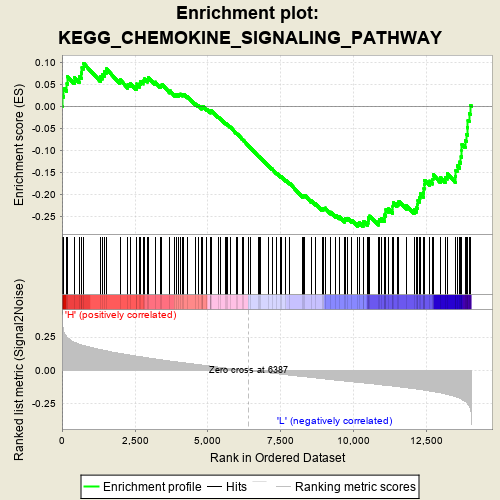

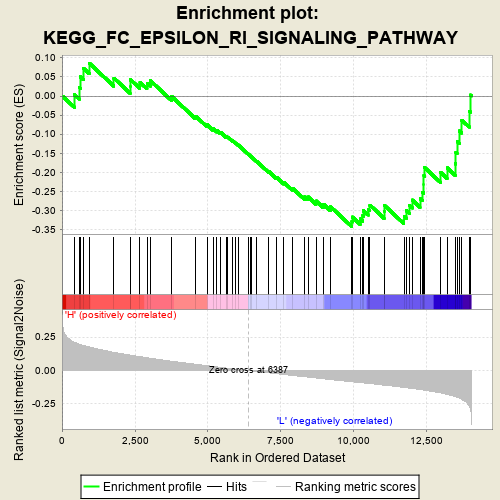


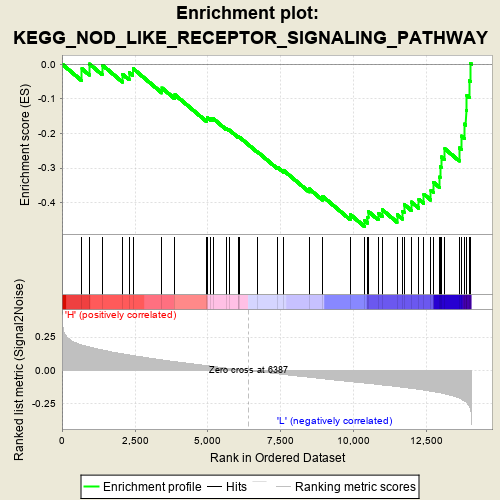

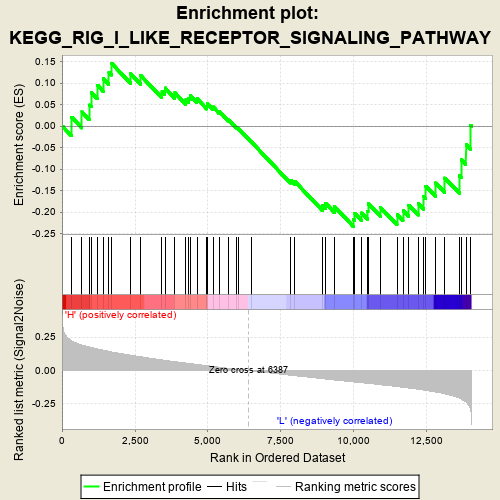


**SM Figure 9: GSEA for promoting immunity related pathways in validation cohort.**

The results indicated that Fc epsilon RI signaling pathway, chemokine signaling pathway, RIG-I-like receptor signaling pathway and NOD like receptor signaling pathway are significantly suppressed immunity-A endotype. GSEA: Gene Set Enrichment Analysis.
